# Mechanical Strain, Temperature, and Misalignment Effects on Data Communication between Piezoceramic Ultrasonic Transducers

**DOI:** 10.3390/s24175561

**Published:** 2024-08-28

**Authors:** Isabel Giron Camerini, Luis Paulo Brasil de Souza, Paula Medeiros Proença Gouvea, Arthur Martins Barbosa Braga

**Affiliations:** Department of Mechanical Engineering, Pontifical Catholic University of Rio de Janeiro (PUC-Rio), Rio de Janeiro 22451-900, Brazilpgouvea@puc-rio.br (P.M.P.G.); abraga@puc-rio.br (A.M.B.B.)

**Keywords:** ultrasonic telemetry, elastic wave propagation, piezoceramic transducers, ultrasonic communication

## Abstract

Acoustic waves can be used for wireless telemetry as an alternative to situations where electrical or optical penetrators are unsuitable. However, the response of the ultrasonic transducer can be greatly affected by temperature variations, mechanical deformations, misalignment between transducers, and multiple layers in the propagation zone. Therefore, this work sought to quantify such influences on communication between ultrasonic transducers. The experimental measurements were performed at the frequency where power transfer is maximized. Moreover, there were four experimental models, each with its own performed setup. The ultrasonic transducers are attached to both sides of a 6 mm thick stainless-steel plate for configuring just one barrier. Multiple layers of transducers are attached to the outer side of two plates immersed in an acoustic fluid with a 100 mm thick barrier. In both cases, the S21 parameter was used to quantify the influence of the physical barrier because it correlates with the power flow between ports that return after a given excitation. The results showed that when a maximum deformation of 1250 μm/m was applied, the amplitude of the S21 parameter varied around +0.7 dB. Furthermore, increasing the temperature from 30 to 100 °C slightly affected the S21 (+0.8 dB), but the signal decayed quickly for temperatures beyond 100 °C. Additionally, the ultrasonic communication with a multiple layer was found to occur under misalignment with an intersection area of up to 40%. None of the factors evaluated resulted in insufficient power transfer, except for a large misalignment between the transducers. Such results indicate that this type of communication can be a robust alternative, with a minimum alignment of 40% between transducers and electrical penetrators.

## 1. Introduction

In some situations, electrical or optical communication between two media separated by a wall can not be accomplished by feeding through cables or connectors. One example that comes to mind is when trying to exchange data and power with sensors or actuators placed inside a sealed vessel that cannot be perforated due to health and safety risks. In this and other cases, electromagnetic or acoustic coupling may provide the required cableless telemetry, transferring data and energy through a channel formed by one or more layers of solids and intercalated fluids. A thorough review by Yang et al. [1] discusses both classes of wireless communication, pointing out that, when compared to different methods of electromagnetic coupling, the use of ultrasonic systems for that purpose brings gains in both efficiency and data rate.

Even though its first related patent was filed in 1997 [2], wireless transmission of power and data through a wall using ultrasonic waves is still a relatively new method in the industry. In its simplest configuration, a pair of concentrically aligned ultrasonic transducers (UTs) are mounted on the opposite sides of a metallic wall. In most applications, these ultrasonic transducers are piezoelectric ceramics that emit and receive ultrasonic signals. Moreover, the system must be resonant to be efficient, and the best impedance match between emitters and receivers must always be sought. However, changes in ambient temperature, mechanical stresses, and strains generated in the wall, as well as misalignment between the ultrasonic transducers, tend to affect the quality of the communication through the channel. Nevertheless, as can be inferred from the ensuing literature review, although these effects have been separately studied, there are still questions regarding the severity of their combined effects. We address those in the present contribution.

Hu et al. [3] have theoretically demonstrated the feasibility of transmitting energy through a metallic barrier using piezoceramic transducers. Following their work, Sherrit et al. [4] employed numerical simulations to investigate how dielectric, piezoelectric, and mechanical losses in the system affect its transfer efficiency, concluding that the adhesive layers that couple the UTs to the wall must play an important role in the overall system response. In an ensuing article, Sherrit et al. [5] explored their prior results to examine three different options for bonding the UTs into the faces of the wall. Their experiments with conductive epoxy demonstrated a 40% efficiency in power transfer. They also analyzed nonlinear and thermal effects, proposing an operational envelope for their specific design. Sherrit et al. [6] reported the design and characterization of a prototype for an acoustic-electric-feed-through capable of transferring 1 kW, with an efficiency of 88%, through a 5 mm thick titanium plate via ultrasonic waves. Other authors explored the subject and reported experimental results for transmission through single metallic walls with thicknesses spanning from 1.6 to 304.8 mm, using a diversity of types and sizes of ultrasonic transducers at different ranges of excitation frequencies (e.g., [7,8,9,10,11]), and reaching close to 60% efficiency, transferring over a 100 W of power [12] with data rates of the order of hundreds of Mbps [13].

Freychet et al. [14] explored using a single transmitter UT with one of its electrodes divided into multiple patches. They aimed to demonstrate using a multiple-input single-output (MISO) strategy to focalize the input ultrasonic waves onto the receiver, increasing power efficiency and improving the robustness of the system. Using a UT receiver smaller than the electrode-etched transmitter, they demonstrated that the system’s response improved whether both transducers were aligned or not. Freychet et al. [15] also employed a piezoelectric stack as the transmitter UT, with all layers in an aligned polarization and a single-layer receiver. They demonstrated that this configuration improved the normalized transmitted power and the power density of the system. Allam et al. [16] present a completed system for data transmission through a metal barrier. The paper describes its behavior through experiments and analytical and numerical modeling. The numerical simulations obtained results close to the experiments, and it was also possible to deliver 17.5 W of DC power to a resistive load through a 3 mm aluminum barrier with an efficiency of 68%. Later, Allam et al. [17] worked on the problem of data and power transfer through detached metal layers. The work focused on the issue of the coupling fluid that influences handling, so Allam et al. proposed soft elastomers for dry coupling, which facilitate the detachable transducers. As a result, using the soft elastomer allowed the delivery of 3 W DC of power with an efficiency of 50% through a 3 mm thick aluminum plate with gaps of 0.5 mm.

Chakraborty et al. [18] extended the analysis from a single wall to a three-layer configuration consisting of two plane metallic walls, respectively, 15.97 and 10.92 mm thick, surrounding an intermediate 88.3 mm fluid layer. Two identical, 25.4 mm diameter, 1 MHz piezoceramic discs were concentrically coupled to the opposite sides of each metallic wall surrounding the fluid layer. The discs were bonded to the walls by using an epoxy-based adhesive layer. Their experiments yielded a data transmission rate of 4 Mbps with power transfer efficiency better than 30%. Furthering the investigation on multi-layer acoustic channels, Takahashi et al. [10] designed and performed experiments on a curved, three-layer system formed by two concentric metallic pipes with a fluid-filled annulus. Their goal was to investigate the wireless transmission of power and data from the production tubing of an oil well through an annulus filled with a water-based fluid and across the casing pipe wall to access sensors placed behind the casing string. To isolate the piezoceramic UT from the annular fluid, they were coupled, using an epoxy resin, to the inside face of a metallic cover that played the role of a first wall. A MEMS-based temperature and pressure sensor was successfully powered and interrogated through all the layers, with a power insertion loss of 10.72 dB and a data transmission rate of 1.2 kbps using an amplitude modulated scheme with Manchester coding. Pereira et al. [11] demonstrated utilizing a pair of co-axially aligned piezoelectric transducers to communicate power and data through two steel plates separated by a fluid layer. The authors employed a frequency shift key (FSK) scheme with automatic gain and carrier control, significantly reducing energy consumption and improving the data transfer error rate. With an overall consumption of 1.2 W, they could transfer 66 mW of power through two flat steel plates (5 mm) separated by a fluid layer (100 mm) at 19.2 kbps. To enhance communication, Zhang et al. [19] present a solution for echo cancellation. This innovative approach, a blend of hardware and a deep learning algorithm, leverages the DPRNN (Dual-Path Recurrent Neural Network) to improve signal quality through a 50 mm aluminum metal barrier. The potential impact of this solution is significant, with a 20 dB increase.

Temperature variations are one of the main factors that affect the propagation of ultrasonic acoustic waves [7,20,21]. Hence, characterizing and investigating the efficiency of ultrasonic communication systems under the influence of such factors is of great importance to developing the ultrasonic telemetry field. Recent works have disclosed ultrasonic wave-based communication systems that were proven to be feasible at different temperature conditions (e.g., [22,23,24,25]). Fishta et al. (2022) [22] present the design of a communication system based on the propagation of acoustic waves in an urban water distribution pipeline. The frequency response of the system is modeled in a black-box approach, and the derived model is used to simulate a complete communication system. The authors show that a good choice of modulation parameters is critical in dealing with problems like frequency selectivity and multipath propagation. Their communication tests demonstrated successful transmission while the environmental temperature varied from 5 to 17 °C. However, such a temperature range lies in an interval that reflects natural ecological conditions. Other works by Huang et al. [23] investigated the efficiency of ultrasonic communication systems under more extreme conditions. They investigated the influence of temperature on communication systems based on ultrasonic guided waves.

A steel pipe was instrumented with LiNbO3 transducers and a temperature controller to evaluate the communication system, ranging from room temperature to 150 °C. The signal-to-noise error (SNR) and the bit error rate (BER) were the metrics for estimating communication performance. According to the authors in [23], the ultrasonic communication system is feasible at least to 150 °C, reaching an error-free bit rate of 10 kbps. Heifetz et al. [24] investigated the feasibility of using a nuclear-grade stainless-steel pipe as a channel to transmit image data with ultrasonic elastic waves. Heating tapes, temperature controllers, and thermal insulation were installed on a laboratory-scale replica of a stainless-steel pipe found in a chemical volume control system to simulate nuclear facility conditions at high temperatures. An amplitude shift keying protocol combined with a root-raised-cosine filter enhanced data transmission compared to square pulse encoding. The results indicated that a 90 kB image could be transmitted with a 10 kbps bit rate and BER of $10^{-3}$ across a six-foot pipe at 230 °C.

Another factor that affects the propagation of ultrasonic waves is the action of mechanical stresses in the acoustic channel. The acoustoelastic effect, i.e., the influence of the stress field on the propagation of elastic waves in elastic media, has been thoroughly investigated for many decades and proven to be an efficient stress measurement technique [26,27,28]. However, it may be detrimental in ultrasonic telemetry by interfering with the impedance of parts that make up the acoustic channel. However, most works investigating the influence of mechanical stress on ultrasonic waves refer to non-destructive testing (NDT) or structural health monitoring (SHM) applications. Regarding the latter, the interest in employing active, piezoceramic pairs of transmitter-receivers to detect faults (continuously and in real-time) in structures dates from the early 1990s, and the field of SHM has grown considerably since then. In these applications, transmitters and receivers are permanently attached to the monitored structure, and, therefore, the influence of stress/strain variations in the ultrasonic channel may be more pervasive than in NDT practice. Giurgiutiu, in [26], emphasizes this point by advocating a combined, predictive approach to improve the reliability and probability of detection of piezoelectric wafer active sensors (PWAS), where environmental changes, underlying vibration, and manufacturing variability are jointly taken into accounting. Currently, several advances in the study of wave propagation in materials subjected to deformation and temperature conditions have been taking place, as in Wang et al. [29] and Zhang et al. [30]. In both works, the study is not focused on the transmission of data or energy but rather on the influence of such physical factors on the ultrasonic wave, which shows the scope of the research.

Misalignment is also a detrimental factor for ultrasonic communication through walls, as mentioned by Chase et al. [31] and Takahashi et al. [10], who chose to use larger UTs and avoid such effects. Ashdown et al. [13] show, in an experimental form, the decay of the magnitude of the response when the transducers are not aligned in the common structural sandwich model consisting of UT–steel wall–UT.

As the literature review demonstrates, recent works have primarily focused on individual factors that influence the propagation of ultrasonic waves in communication systems. However, the combined influence of mechanical stresses, temperature, and alignment has been largely unexplored. This is the gap that our research aims to fill. We meticulously designed four experimental test models to study these factors comprehensively from different perspectives. Our work is dedicated to analyzing the variation in parameter S21, a key relationship between the input and output signals. To conduct this analysis, we developed specimens and subjected them to temperature variations and mechanical deformation close to yield strength. Additionally, we thoroughly analyzed ultrasonic communication under the condition of multiple layers (with the addition of water in the middle) and the influence on the transmission according to the intersection area, varying the alignment of the transducers.

## 2. Theoretical Approach

### 2.1. Acoustoelasticity

The acoustoelastic effect is identified in tensile tests monitored with ultrasound [32]. It studies the variation in the velocity of elastic waves when the body is subjected to stress [5,27]. When this effect is considered in ultrasonic measurements, one must assume that there is a nonlinear relation between the speed of elastic waves and the stress field in the solid media. Compared with other ultrasonic waves, longitudinal waves have the highest sensitivity for applications under mechanical stress [33]. However, longitudinal waves are relatively small and decay very fast, making the acoustoelastic effect a small effect [33,34].

For example, in metals, mechanical stress causes a variation in the ultrasonic velocity of only 3%, which is considered a small variation in the measurement but may generate large errors in measurements based on time-of-flight (TOF) techniques. Another issue is that other external factors, such as temperature, coupling conditions, anisotropic properties of materials, and micro-textures, among other factors, also influence ultrasound waves. Of those listed, temperature and coupling conditions have been the main factors affecting the measurement of mechanical stress [27,35].

Hughes and Kelly [36] initially developed the theory of acoustoelasticity and its modeling. Previously, the effect was associated only with perfectly elastic solids. The deformation was a function of mechanical stress and temperature, and the model was based on infinitesimal deformations. The modern theory of acoustoelasticity is completed with the general theory of finite and nonlinear deformations presented by Murnaghan [36]. For this effect to be associated only with elastic linear strains, the model is based on Hooke’s law, which is the linear relationship between stress and strain [37]. However, this model fails to describe the relationship between mechanical stress and acoustic waves observed in practice [32].

Practical observations clarify that when acoustic waves propagate in metal, they cause the displacement of particles that superpose the existing internal tensions. Considering the linear elastic relationship methodology, the solution would be just overlapping waves without one influencing the other. After crossing, they would continue traveling without affecting one another [32]. However, this is not what happens in the acoustoelastic case. Mechanical stress influences the acoustic wave, and the experimental model can only be explained mathematically by introducing a nonlinear elastic model of finite deformations [32,37], i.e., the stress–strain relationship can be linear. However, it becomes necessary to use nonlinear strain and stress related to linearity, an equivalent stiffness tensor for the dynamic deformation of waves [38], making the formulation require second- and third-order coefficients [35,39].

### 2.2. Piezoelasticity under the Influence of Strain and Temperature Variations

Ultrasonic transducers of the piezoelectric ceramic types play a crucial role in this work, as they propagate ultrasonic waves [40]. These transducers convert a short electrical pulse in the UT into acoustic waves. This conversion is made possible by the unique property of piezoelectricity, found in certain crystalline materials like quartz and perovskite and ceramics based on barium titanate, lead zirconate titanate, or PZT [41,42]. These ceramics, belonging to the ferroelectric materials class, exhibit a spontaneous polarization in a temperature range. This polarization can be reversed with the application of an external electric field. However, they are isotropic when newly produced and present a macroscopic orientation of spontaneous polarization [42,43]. For this reason, to be used as piezoelectric sensors, they need to be polarized under the application of high electric fields (kV/mm) at a high temperature, around 90 °C, so it is possible to choose the direction of the macroscopic polarization. This versatility allows for various applications, from medical imaging to industrial testing. Thus, the main characteristic of piezoelectric ceramics is that they present an electrical polarization when the material is subjected to deformation or mechanical load. In other words, electric charges and potentials arise when applying mechanical stresses and strains. This phenomenon is called the direct piezoelectric effect. It is also observed that the applied electrical energy is transformed into mechanical energy, called the inverse piezoelectric effect [41].

Piezoelectric materials are exhibited with about 20 to 32 classes of crystals. They are always associated with non-center-symmetric crystals [44], with the mechanical relation being symmetric and the electrical connection being antisymmetric. All piezoelectric effects occur in materials that have an asymmetric lattice in such a way that it is possible to produce electric dipole moments while having some deformation. Therefore, all piezoelectrics are anisotropic [45]. In addition, PZT transducers have specific natural frequencies associated with their elastic properties and geometry [46]. The natural frequency $\omega_{n}$ for undamped systems is a relation between the stiffness and the inertia of the system. In under-damped vibration systems, the natural under-damped frequencies $\omega_{d}$ are harmonic responses as a function of the undamped natural frequency $\omega_{n}$. The damping factor ζ is represented in Equation (1) [47].

(1) $$\omega_{d}=\omega_{n}\sqrt{1-\zeta^{2}}$$

The ζ is the damping factor, a dimensionless variable with a value of 0≤ζ≤1. The closer ζ is to 1, the greater the damping, which means the faster the system energy will dissipate [46]. In addition to this basic concept for natural frequencies, it is known that piezoelectric ceramic disks vibrate basically in two fundamental modes, the radial mode and the longitudinal mode [48]. The radial mode generates mechanical vibrations mostly in the radial direction. In contrast, the longitudinal mode generates mechanical vibrations mainly in the longitudinal direction.

**Figure 1:**
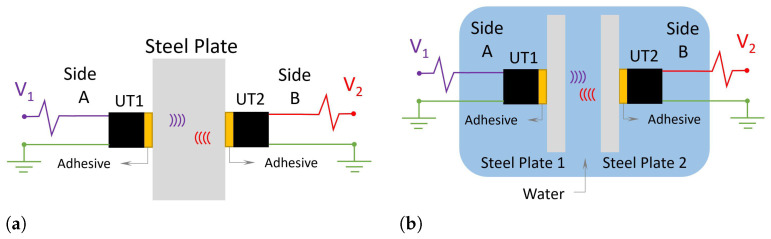
Illustration of the propagation of acoustic waves through the pitch-catch technique for monolayers and multilayer of materials. (**a**) Conf.1, where the ultrasonic transducers (UTs) are on opposite sides of the same stainless-steel plate. In this configuration, the ultrasonic wave travels only through stainless-steel. (**b**) Conf.2, in this case, the pair of ultrasonic transducers are on different stainless-steel plates, but they are also aligned horizontally, and there is fluid between them.

Several techniques are used to collect the ultrasonic response, such as pitch-catch [26]. In pitch-catch procedures, separate UT transmitters and receivers are used, each in a middle zone. The UT transmitter transforms the electrical pulse into an ultrasonic wave propagating through the material. The UT receiver—preferably as aligned as possible with the UT transmitter—performs the reverse process, transforming the ultrasonic wave into an electrical pulse [40]. The physical model used for data transfer is illustrated in Figure 1.

Figure 1a presents the model used in the tests, in which an electrical pulse, named *V*, excites one of the UTs coupled to the steel plate or the steel-water-steel assembly (Figure 1b). This UT transforms the electrical signal pulse into an ultrasonic wave propagating through the materials. After passing through this entire region, the receiving UT will receive this ultrasonic wave and transform it into an electrical signal [10,49].

The amount of energy transmitted between the transducers depends on several factors, such as the shape of the excitation signal and the excitation frequency [48,49]. Energy transmission is improved as the transducer is excited close to its resonant frequency.

In the case in question, the two transducers will be excited by consecutive sinusoidal pulses that sweep in frequency through a previously determined window in Hz, according to the resonant frequency of interest. The signals emitted by the transducers varying in frequency are called mirroring parameters S11 and S22, which, in the case of this research, are excited with an interval of 3 ms from one to the other. This research involves a complex analysis, allowing for examining the maximum energy through the valley of mirroring parameters S11 or S22 in power transmission and communication systems between UTs. The valley of curves S11 and S22 is where the highest energy transmission occurs. This value is also the value where the transition impedance is maximized. The analysis of parameters S21 and S12 allows for the analysis of the maximum energy transmitter through the valley of mirroring parameters S11 or S22 in power transmission and communication systems between UTs, allowing a quantification of the energy transmission through them. It is necessary to understand the communication between the transmitter and the receiver. This communication can be characterized by the parameter S21 when comparing the response of UT1 as a transmitter and UT2 as a receiver or by S12 when UT2 is the transmitter and UT1 is the receiver.

For this reason, analyzing the maximum energy through the valley of mirroring parameters S11 or S22 in power transmission and communication systems between ultrasonic transducers is important. This transducer–receiver relationship is given by the logarithmic ratio between the Fourier transform of the voltage of the UT receiver (V2) and the Fourier transform of the voltage of the UT transmitter (V1). This relationship is expressed in Equation (2).

(2) S21=20logF[V2(t)]_F[V1(t)]

Signals S11, S22, S21, and S12 are provided by equipment called vector network analyzers (VNAs), whose properties and characteristics are described in Section 3.

External factors such as temperature variation, mechanical deformation, and transducer alignment directly influence the ultrasonic wave. The temperature variation of the environment where the system is installed directly affects the speed of wave propagation [35]. It has emphasis because it changes on a large scale in open and inhospitable environments and significantly varies the wavelength [20]. Generally, the effect of temperature is measured and empirically removed. However, certain consequences of the temperature variations are not considered when doing this. For example, the UT can compress or lengthen the response of the signal or even change the appearance of the signal [50]. The advantage is that the longitudinal wave velocity can be related as expressed in Equation (3) [35].

(3) $$v_{l}=k_{l}\Delta T+v_{lT_{0}}$$

where $v_{l}$ is the longitudinal wave velocity, $k_{l}$ is the temperature sensitivity of the longitudinal wave velocity, ΔT is the temperature variation about the reference temperature $T_{0}$, and $v_{lT_{0}}$ is the longitudinal velocity for the reference temperature.

For mechanical strain, the elastic properties of elastic waves are directly influenced by the applied mechanical stress. However, the phase velocity is related to the elastic constants and the stress of the material [27,35]. The speed of the longitudinal wave is Equation (4), and the stress can be represented as the force *f* by the surface area on which it is applied, as in Equation (5). Alternatively, the stress can also be derived based on Hooke’s law, which relates the applied stress to the deformation [37], as represented in Equation (6). In this case, the stress $\sigma_{ij}$ is the product of the strain ($\varepsilon_{ij}$) and the matrix of elastic constants of the material.

(4) $$v_{l}=\sqrt{\frac{\lambda +2\mu }{\rho_{0}}}$$

(5) $$\sigma_{ij}=\frac{f}{A_{s}}$$

(6) $$\sigma_{ij}=C_{ijkl}\varepsilon_{ij}$$

The strain ($\varepsilon_{ij}$) mentioned in Equation (6) can be described by a variation in area A, as in Equation (7).

(7) $$\varepsilon_{ij}=\frac{\Delta A}{A_{0}}$$

Relating the equations from Equation (4) to Equation (7), it is possible to obtain the equation for the uni-axial tension with the velocity of the longitudinal wave, shown in Equations (8) and (9).

(8) $$\rho v_{l_{x}}^{2}=\lambda +2\mu +\frac{\sigma_{x}}{3\lambda +2\mu }\left[2l+\lambda +\frac{\left(\lambda +\mu \right)\left(4m+4\lambda +10\mu \right)}{\mu }\right]$$

(9) $$\rho v_{l_{y}}^{2}=\lambda +2\mu +\frac{\sigma_{x}}{3\lambda +2\mu }\left[2l-\frac{2\lambda }{\mu }\left(m+\lambda +2\mu \right)\right]$$

**Figure 2:**
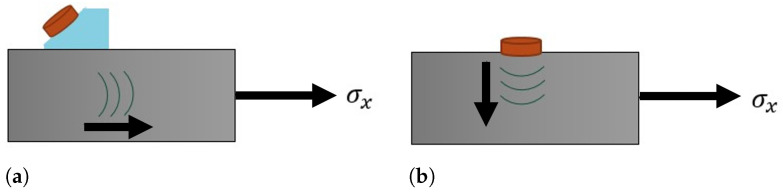
Schematic of the wave propagation. (**a**) Wave propagation parallel to the applied stress, i.e., stress *x*-axis ($\sigma_{x}$) and $v_{l_{x}}$; (**b**) Wave propagation perpendicular to the applied stress, i.e., stress *x*-axis ($\sigma_{x}$) and $v_{l_{y}}$.

In Equations (8) and (9), the λ and the μ are second-order Lamé elastic in isotropic media, and *l* and *m* are third-order elastic constants, also known as Murnaghan constants. Equation (8) is the equation for tensile stress acting on the *x*-axis ($\sigma_{x}$) and the propagation of the ultrasonic wave also parallel to the *x*-axis, as represented in Figure 2a. Equation (9) is the equation for tensile stress. In Figure 2b, the applied tensile stress is also in the *x*-axis, but the propagation of the ultrasonic wave is now perpendicular to this stress, being valid for $v_{l_{y}}$ and $v_{l_{z}}$ [51].

As mentioned earlier, this work uses the pitch-catch model and a traditional tensile test. The propagation of the wave will be perpendicular to the applied voltage. Therefore, the configuration used will be that of Equation (9) and Figure 2b.

## 3. Experimental Methodology

The analysis of ultrasonic communication was performed using a diverse range of tests including temperature variation, stress tensor test, stress tensor test with temperature variation, and the UT misalignment test in a multiple barrier configuration. In addition, these temperature tests were also performed with the transducer in different positions and with fluid in the middle. These four types of tests led to the development of two models of specimens, called SP.1 and SP.2. Both were developed in AISI 316 stainless-steel and have a chemical composition of 16% chromium, 10% nickel, and an additional addition of 2% molybdenum. This combination, which is among the most common and usual, is classified in the austenitic family [52].

**Figure 3:**
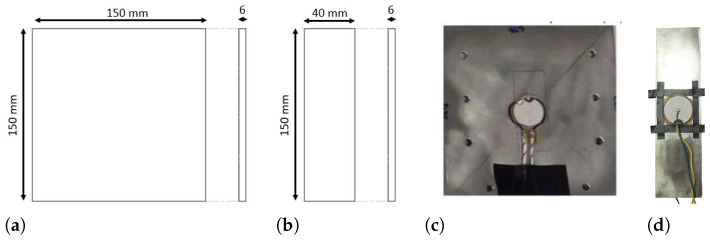
The two models of specimens used in the 4 test models carried out were made in stainless-steel. (**a**) SP.1 dimensions: this specimen is used in the Test Models 1 and 2, in Conf.1 and Conf.2. (**b**) SP.2 dimensions: this specimen is used in the Test Models 3 and 4 and only in Conf.1. (**c**) Photograph of SP.1 with the ultrasonic transducer coupled; one can also observe the electrical wires already soldered and side holes where the sealing box will be attached for Conf.2. (**d**) Photograph of SP.2 with the ultrasonic transducer coupled; one can also observe the electrical wires already soldered.

The dimensions of SP.1 are presented in Figure 3a. This specimen model is used in the temperature and temperature tests with variations in the positioning of the plates with fluid in the middle. Figure 3b shows the dimensions of SP.2 used in the strain tests. The cross-section of SP.2 is 40 mm × 6 mm, which results in a cross-sectional area of A = 240 mm^2^. The Young’s modulus for this stainless-steel is 200 GPa, so by applying this to Equation (5), it is possible to deduce that the maximum force in the specimen not to occur plastic deformation is 69.9 kN. This results in a yield strength of 291 MPa and a strain of 1456 μm/m. Furthermore, the SP.2 specimen was designed for the size of the ultrasonic transducer. However, due to limits in the geometry of the Instron machine used in the tests, this did not follow the standard norm for traction tests ASTM E8/E8M-9 [53].

**Figure 4:**
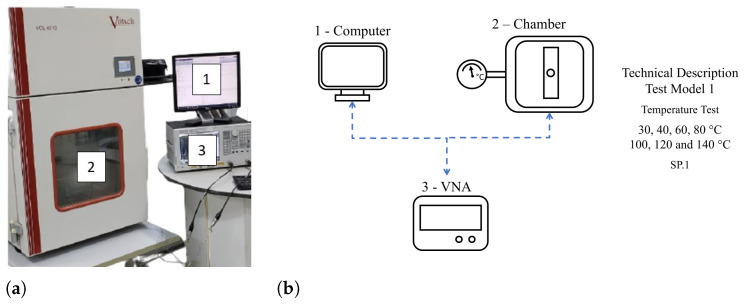
Schematic of the Test Model 1—the temperature variation test. (**a**) Photograph of the temperature variation test bench. (**b**) Test Model 1 diagram. (1) The test controller computer; (2) the climatic chamber in which the specimen is allocated; and (3) the vector network analyzer which sends and receives signals S21.

The test was carried out in two different configurations of data transmission. In the first one, Conf.1, the ultrasonic transducers are fixed and aligned on opposite sides of the specimen. The energy is transferred horizontally through the propagation medium, which is the body of the specimen, as shown in Figure 1a. In the other configuration (Conf.2), two ultrasonic transducers are fixed on different plates, submerged parallel in a fluid that forms a fluid barrier between them. The propagation media are the two stainless-steel plates and the fluid layer in the middle, as illustrated in Figure 1b. In Conf.1, a climatic chamber was coupled to the setup test. The climatic chamber is from Vötsch (model VCL4010), depicted by number 2 in Figure 4a. This equipment allows temperature control from −40 to 180 °C, with a variable rate of 3 °C/min and stability of ±0.5 °C. Test programming is carried out and controlled by the climatic chamber itself. In Conf.2, the setup was immersed in a thermal bath (Lauda, model Alpha RA 24), which heated the fluid in a controlled manner varying from −25 °C to 80 °C and stability ±0.05 °C and was used to maintain the same temperature—30 °C. A mobile support was made that allowed the plates to maintain a vertical position even when immersed in the thermal bath.

The VNA model E5061B from Keysight, is a versatile tool that can adapt to various measurement needs. It sends and reads the UT waves and can measure impedance in a frequency range from 5 Hz to 3 GHz. According to the specifications of the manufacturer, it has a wide dynamic range, around 125 dB, a measurement speed of 3 ms, a trace noise of 0.0005 dBrms, and temperature stability of 0.005 dB/°C. The UTs are excited by consecutive sinusoidal pulses sweeping in frequency through a previously determined Hz window. The VNA sends this frequency range for the UT according to the piezoelectric ceramic (PZT) resonant frequency, which in this case is 1 MHz. The signals emitted by the transducers varying in frequency are called mirroring parameters or S11 and S22, in the case of the simple structural sandwich with only two UTs. If they have more UTs, more mirroring parameters are available; for each UT combination, there will be two mirroring parameters. The VNA has the function of sending a pulse for multiple PZTs, which receive the same excitation. However, in the propagation environment, the wave propagates at different speeds. This fact occurs due to variations in the resonance frequency that can be generated due to external factors, such as poor adhesion of the system, the appearance of bubbles, or some variation in the geometry or property of the material. For this reason, analyzing parameters S11 and S22 individually for power transmission systems and communication between UTs is insufficient because it is necessary to understand this mirroring parameter transducer–receiver, so the parameter of interest to be analyzed in this article is S21 because it relates to the sender (1) with the receiver (2).

Another consideration about this S21 mirroring parameter is the valley of the curves, where the highest energy transmission occurs. This value also maximizes the transition impedance; temperature variation, mechanical deformation, and the horizontal non-alignment of the transducers influence its variations and alternations. The VNA receives and sends signals S11, S22, and S21, and the equipment is connected by electrical cables previously welded in the PZT. To carry out the communication and data acquisition, a computer with MATLAB® is used to pre-process the data together with the VNA.

Experimental validation was carried out in four experiments. Test Model 1 is a temperature variation test only. The temperatures of this test model were applied in a cycle between temperatures from 40 °C to 140 °C with steps at intervals of 20 °C, at which the temperature was kept fixed for two hours. The temperature interval was chosen according to the polarization of the PZT; according to literature data, above 80 °C, the PZT begins to depolarize [54,55]. However, preliminary tests indicated that the amplitude of the S21 signal was optimized up to 100 °C. After this temperature, the parameter deteriorated in its response. For this reason, the tests were carried out with temperatures up to 140 °C. Figure 4a shows the experimental bench for this test, and the schematic for the experiment and its properties are shown in Figure 4b.

**Figure 5:**
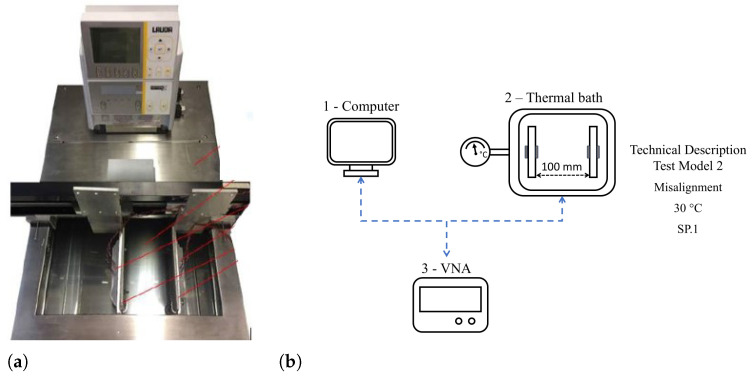
Schematic of the Test Model 2—the misalignment test. (**a**) Photograph of the thermal bath. (**b**) Test Model 2 diagram. (1) The test controller computer; (2) the thermal bath in which the specimen is allocated; and (3) the vector network analyzer which sends and receives signals S21.

Test Model 2 is a test immersed in the thermal bath with the fluid barrier, where the transducers have horizontal linearity variations of 100%—fully aligned—to 0% misaligned. The horizontal linearity test of the plates (Model 2 test) was set to maintain a fluid barrier distance of 100 mm between them. The bath temperature was fixed at 30 °C ambient temperature. Figure 5a shows the thermal bath used, and it is possible to observe the two SP.1 plates in parallel inside the thermal bath. Figure 5b is the Test Model 2 schematic and the technical description.

**Figure 6:**
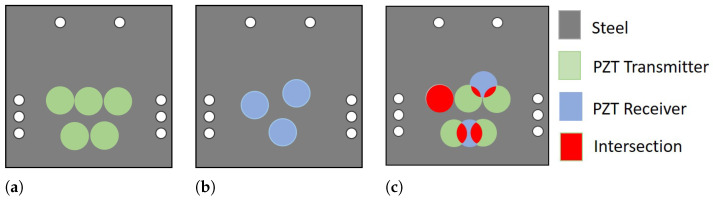
Representation of the instrumentation of the specimens for the alignment test. (**a**) The plate on the left, with five PZTs, is lined up in green. (**b**) Right plate, with three unaligned PZTs in blue. (**c**) The left and right plates overlapped, whereas red was the overlapping area. The white dots represented are spacers for centralizers to align the plates.

For carrying the Test Model 2, eight PZTs were used. Five PZTs were glued in an aligned pattern on plate 1 (in green in Figure 6a), and three PZTs were glued in a misaligned pattern on plate 2 (in blue in Figure 6b). Figure 6c shows the red intersection area between the two plates. The misalignment was calculated to impose intersections of 100%, 40%, 12.5%, and 0%, with 100% completely aligned and 0% completely misaligned.

**Figure 7:**
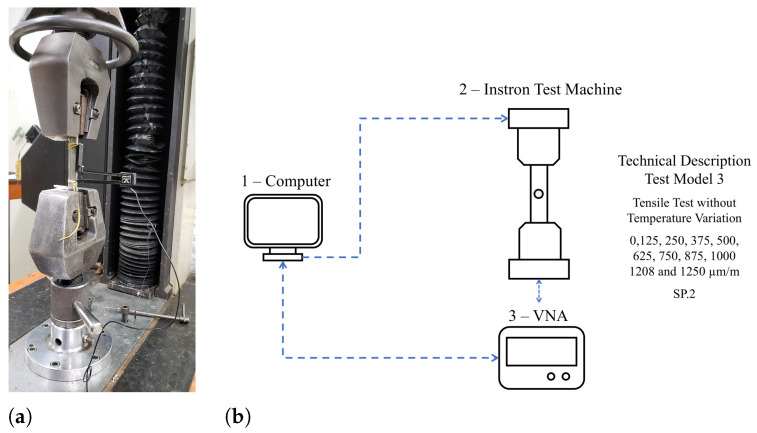
Schematic of the Test Model 3—the tensile test without temperature variation. (**a**) Photograph of the tensile machine Instron, model 8502. (**b**) Test Model 2 diagram. (1) The test controller computer; (2) Instron model 8502; and (3) the vector network analyzer which sends and receives signals S21.

Test Models 3 and 4 are the tensile tests, without temperature variation and with temperature variation, respectively. For this reason, two tensile testing machines were used to carry out the tests. Test Model 3 was performed in a robust servohydraulic tensile machine (Instron, model 8502) (Figure 7a), which withstands stresses of up to 100 kN. In this model, it is not possible to couple the climatic chamber due to the large dimensions of the machine and the limited space available in the climatic chamber. Therefore, the test was performed in this tensile machine to observe the UT communication around the specimen yield strength. According to the properties of the material and the stipulated geometry, this value is MPa, i.e., 1460 μm/m. The maximum load used was 60 kN, generating a stress of 250 MPa, corresponding to 85.8% of the yield stress. This mechanical tension reached a maximum deformation of 1250 μm/m. These deformations were applied in steps. The deformation was fixed for 2 min at each step due to the stability time of the stress tensor and data ultrasonic signal collection. The deformation values were 0, 125, 250, 375, 500, 625, 875, 1000, 1104, 1208, and 1250 μm/m. The Test Model 3 diagram and the technical description are detailed in Figure 7b.

**Figure 8:**
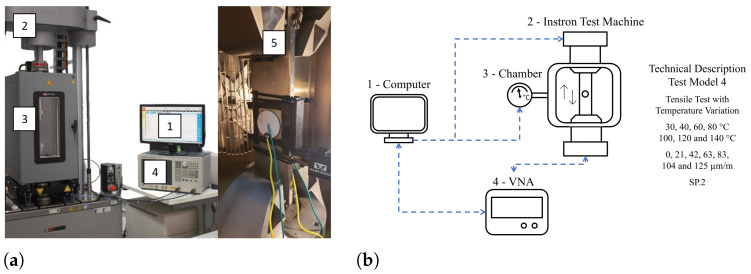
Schematic of the Test Model 4—the tensile test with temperature variation. (**a**) Photograph of the Instron—model E10000; (**b**) Test Model 2 diagram. (1) The test controller computer; (2) Instron model E10000; and (3) the climatic chamber coupled to the Instron machine; (4) the vector network analyzer which sends and receives signals S21; (5) zoom-in on the test specimen inside the thermal chamber coupled to the Instron machine.

The second tensile machine used is an Instron (model E10000) with a climatic chamber operating between −70 °C and 350 °C, temperature stability of ±2 °C, and maximum static stress of 7 kN. The Model 4 tests were carried out in this setup, where ultrasonic communication was possible by applying mechanical strain at different temperatures. This machine only accepts loads up to 7 kN; for safety reasons, the maximum load of this test is 6 kN. According to the geometries of the specimen, this force generates a maximum mechanical stress of 25 MPa and a strain of 125 μm/m, corresponding to 8.62% of the yield strength. As in the Model 3 tests, the stress is also applied gradually during the Model 4 tests. The active strain values were equal to 0, 21, 42, 63, 83, 104, and 125 μm/m, and the temperatures were 40, 60, 80, 100, 120, and 140 °C. Figure 8a shows the photograph of the setup for the Model 4 tests. In the Model 3 and 4 tests, in addition to pre-processing, the control computer also controls the Instron machine, applying the deformation for the indicated time or changing the temperature in the climatic chamber. Figure 8b summarizes, step-by-step, how this interaction is performed. The computer (1) controls the Instron machine (2) with the climatic chamber (3) attached to it, and both are acting on the specimen with the UT coupled (5). The computer (1) also controls the VNA (4), which simultaneously sends and receives the signal to the PZT (5). Finally, it sends it to the computer to save the data, showing a preview (1). The test control logic for the Model 3 test is the same, excluding the climatic chamber. Step (2) would be directly connected with (5). In tests 1 and 2, the support computer only controls the VNA, as both the climatic chamber and the thermal bath are handled by the internal controller of each tool.

Throughout the work, all UT models used a piezoelectric ceramic type 4, the PZT4, shaped like a cylindrical tablet. This kind of PZT has the characteristic of being more rigid than the other piezoelectric ceramic models, which makes this UT perform better as a signal transmitter [37,53]. This PZT is 25 mm in diameter with 1 mm thickness. In addition, the resonance frequency is close to 1 MHz, so to optimize communication, this frequency will be used in the experiments. This PZT also has a “wrap”, which would be a connection between one face and the other by a ceramic band. This band allows the face opposite the face of the wrap to be attached to something and still be welded and excited by this ear.

## 4. Results

### 4.1. Characterization of Signal Variation by Temperature Variation—Test Model 1

**Figure 9:**
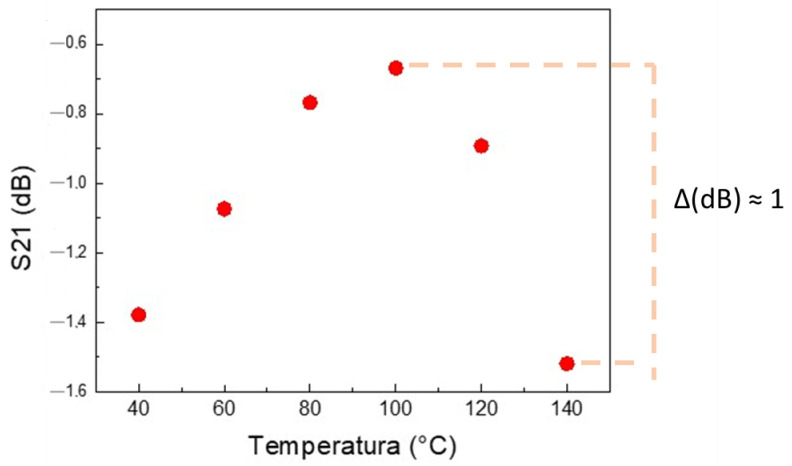
Graph of the values that maximize the signal of S21 for Test Model 1 and SP 1. Each red mark represents the value that maximizes S21 for each temperature. Moreover, the variation in ΔdB is marked on the right side.

The system response for temperatures ranging from 40 °C to 140 °C is shown in Figure 9.

For the temperature range between 40 and 100 °C, S21 increases continuously, from −1.4 to −0.65 dB, with a variation of +0.71 dB, indicating that for this transducer/test specimen set, there is a small improvement in signal transmission up to approximately 100 °C. Above 100 °C, the transmission efficiency between the transducers declines rapidly. However, communication is maintained up to the maximum tested temperature of 140 °C, with a signal decay of approximately −1 dB, as indicated in Figure 9.

It should be noted that this signal remains robust for the proposed application. It presents values that maximize the S21 signal, ranging from −1.6 dB to almost 0.6 dB, which results in excellent communication between the transducers.

### 4.2. Evaluate the Influence of the Intersecting Area between PZTs—Test Model 2

The study meticulously examines the influence of the intersecting area between PZTs on acoustic energy transfer. To ensure the accuracy of our findings, we designed a precise experimental setup. Two parallel metal plates were instrumented with PZTs in specific positions, as per the schematic diagram in Figure 6. These plates were then submerged in water, similar to Conf.2, and positioned at a distance of 100 mm. The intersection areas were varied in alignment percentages from 100%, 40%, 12%, to 0%, with 100% representing perfect alignment and 0% indicating complete misalignment.

**Figure 10:**
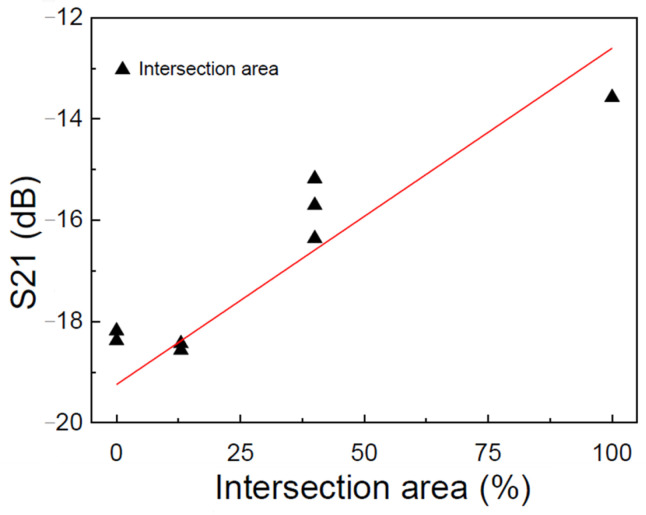
The values that maximize the signal of S21 for the Test Model 2 for SP.1 and Conf.2, where there is a water barrier between the test specimens, are identified by each black mark. These marks represent the value that maximizes S21 for each intersection percentage, guiding us in finding the optimal signal.

These aligned regions, a key aspect of our study, are visually represented in Figure 6. Furthermore, Figure 10 provides a clear visual of the variation in the S21 signal for the other intersections, enhancing understanding of the results.

The first thing to observe in Test Model 2, compared to Test Model 1, is the decay in the S21 signal when the water barrier is added. The signal close to −1 dB before is now −13 dB for the region with the intersection of 100%, as shown in Figure 10. This value for parameter S21 is considered a not-so-good value because the closer to 0, the better the relation between S11 and S22. However, it is still possible to observe the signal transfer with the communication between transmitter and receiver, even with two steel barriers and a large water barrier. These factors are interesting as they indicate that we still have communication between the transducers, proving the data transmission theory with more than one physical barrier, which in this case, is three physical barriers.

Another consideration is the intersection of areas. Communication between transducers is optimized when the transmitter and receiver are perfectly aligned horizontally. The data in Figure 10 show that the signal decreases as the intersection decreases. Parameter S21 is close to −13 dB at the 100% intersection. For the 40% intersection, it is around −15 dB. However, there is still communication between the transducers. For the 12.5% intersection, the signal starts to diffuse close to −19 dB, and for the 0% intersection, the result also maintains this value. The result is then considered no longer to have good ultrasonic communication for these values. In addition, it proves that the more horizontally aligned the devices are, the better the ultrasonic communication and data transmission. The red line on Figure 10 emphasizes a linear relationship between parameter S21 and the intersection of the area, showing a uniform decay of S21 as the misalignment of the transducers worsens.

### 4.3. Tensile Test near to the Yield Strength—Test Model 3

**Figure 11:**
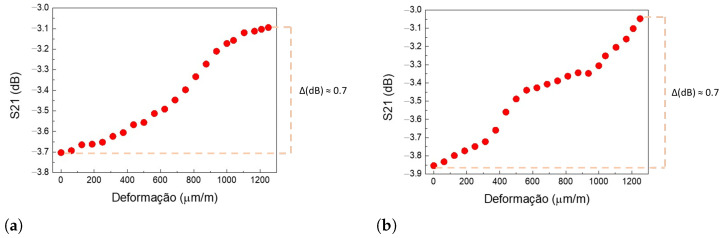
Model 3 test results—values that maximize the parameter S21 (dB) by the mechanical deformation exerted (μm/m). (**a**) Test Model 3-1, where the variation in parameter S21 from the undeformed state to the maximum strain state was approximately 0.7 dB. (**b**) Test Model 3-2, where the variation in parameter S21 from the undeformed state to the maximum strain state was approximately 0.7 dB.

Two specimens of SP.2 were tested in Test Model 3. This test was made with mechanical stresses up to 250 MPa, corresponding to 85.8% of the stainless-steel yield strength and generating a deformation of approximately 1250 μm/m. The graphs in Figure 11a,b show the curve of values that maximize the S21 signal.

Each point on the graphs in Figure 11a,b represents the maximum value of S21 for each strain tested. From the undisturbed condition to the highest strain applied, 1250 μm/m, in both specimens, the variation at the peak of S21 was positive and around 0.7 dB (+0.67 dB for Test Model 3-1 and +0.71 dB for Test Model 3-2). This positive variation indicates that even for higher deformations and mechanical stresses close to the yield strength of the specimen, the power transfer between the transducers remained satisfactory, with S21 between −3.9 and −3.0 dB. The similar response of both tests to the same strain further affirms the performance of the transducers even at high strain.

### 4.4. Tensile Test with Strain and Temperature Varying—Test Model 4

**Figure 12:**
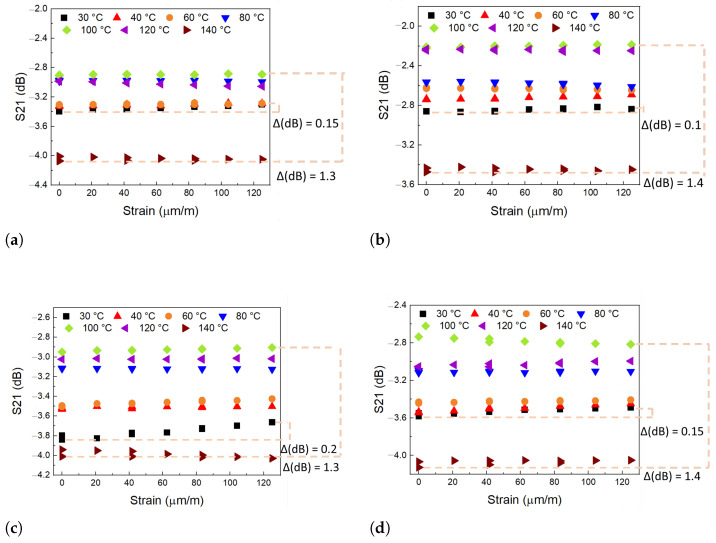
Test Model 4 result—values that maximize the parameter S21 (dB) by the strain (μm/m) and the temperature variation. Each color in the graph represents a different temperature, and each point a different strain. (**a**) Test 4-1: The ΔS21 for the strain influence is +0.15 dB, and the maximum variation in parameter S21 is −1.3 dB for 100 °C with strain and 140 °C without strain. (**b**) Test 4-2: The ΔS21 for the strain influence is +0.1 dB, and the maximum ΔS21 is −1.4 dB for 100 °C with strain and 140 °C without strain. (**c**) Test 4-3: The ΔS21 for the strain influence is +0.2 dB, and the maximum ΔS21 is −1.3 dB for 100 °C with strain and 140 °C without strain. (**d**) Test 4-4: The ΔS21 for the strain influence is +0.15 dB, and the maximum ΔS21 is −1.4 dB for 100 °C with strain and 140 °C without strain.

Test Model 4 is a test with Conf.1 and the SP.2 specimen. The sample is subjected to various mechanical deformations and temperature variations. Four models were tested, with temperature varying up to 140 °C and load up to 6 kN, generating a maximum mechanical stress around 25 MPa, corresponding to a deformation of 125 μm/m, for the SP.2 that has the cross-sectional area of 240 mm^2^. Figure 12 presents the result of the values that maximize parameter S21 for test 4.

The results obtained in the four tests performed on Model 4 showed a variation ΔS21 of approximately 0.15 dB. In all curves, it is observed that the interpretation of S21 with strain is minimal for this range of strains, from 0 to 125 μm/m. The result is compatible with that observed for the specimens tested in test 3. The signal has a small level of hysteresis during the charging cycles, which does not harm the power transmission efficiency between the transducers. All four specimens tested with temperature variation show S21 peak amplitude curves with similar behavior, where 100 °C was the temperature at which the S21 signal had the highest peak values. In the temperature range from 30 to 100 °C, the peak value of the S21 signal changes. For tests 4-1 and 4-2, ΔdB is approximately 0.6; for tests 4-3 and 4-4, this value is about 0.8. In the four cases, the signal decayed sharply after 100 °C and up to 140 °C, varying between 1.2 and 1.3 dB. This variation was a little higher than in the Model 1 test because of the influence of deformation.

It is important to reiterate that in all tests, the peak value of S21 consistently remained between −4.0 and −2.5 dB. The configuration studied here efficiently fulfills the proposed function of interrogating sensors by ultrasonic telemetry through the metal wall. Even in the worst cases, the small variations in S21 of 1 dB do not disrupt the energy transfer and data flow between the transmitter–receiver pair.

**Figure 13:**
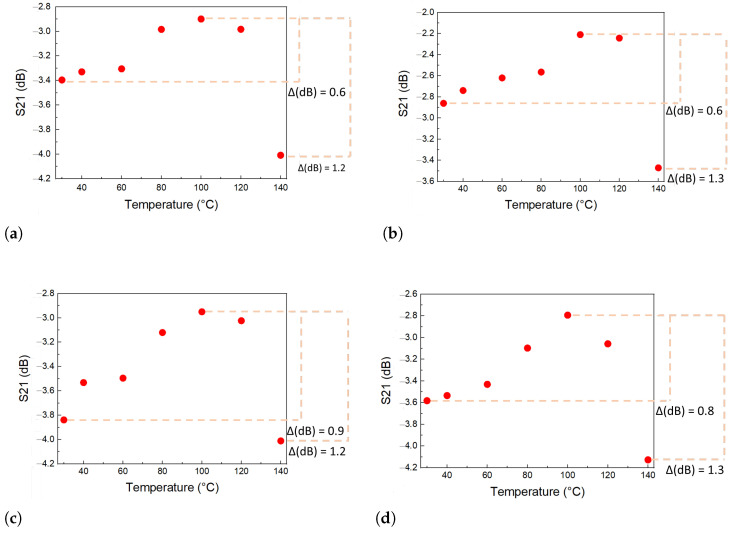
Test Model 4 result: values that maximize the parameter S21 (dB) by the temperature variation compensating for the deformation factor, where each point corresponds to a different temperature. (**a**) Test 4-1: Variation in parameter S21 from ambient temperature to the temperature that maximizes signal S21 (100 °C) is 0.5 dB, and the rapid decay of the parameter after 100 °C to 140 °C, where the ΔdB is 1.2 dB. (**b**) Variation in parameter S21 from ambient temperature to the temperature that maximizes signal S21 (100 °C) is 0.6 dB, and the rapid decay of the parameter after 100 °C to 140 °C, where the ΔdB is 1.3 dB. (**c**) Test 4-3: Variation in parameter S21 from ambient temperature to the temperature that maximizes signal S21 (100 °C) is 0.9 dB, and the rapid decay of the parameter after 100 °C to 140 °C, where the ΔdB is 1.2 dB. (**d**) Test 4-4: Variation in parameter S21 from ambient temperature to the temperature that maximizes signal S21 (100 °C) is 0.8 dB, and the rapid decay of the parameter after 100 °C to 140 °C, where the ΔdB is 1.3 dB.

It is crucial to note that the adhesive undergoes significant changes in its properties, including color alteration and the appearance of a burning odor when exposed to temperatures above 120 °C. This underscores the importance of considering temperature as a significant factor that can influence the performance of the system, as shown in Figure 13.

## 5. Conclusions

The UT used in the test, a piezoelectric ceramic Type 4 in a disk format, 25 mm in diameter and 1 mm in thickness, plays a pivotal role in the experiments. Its natural frequency selected this model to resonate at 1 MHz, the excitation frequency used in the experiments. Far from the natural frequency, it would not allow the signal to propagate perfectly, preventing energy and data transmission. For this reason, the setup is programmed for this resonance window, optimizing the system communication. In the four test models developed, S21 is the analyzed parameter. It is a ratio between the signal emitted by the transmitter and that received by the receiver, and this value is optimized when it is close to zero.

Test Model 1 uses the Conf.1 configuration and the SP.1 specimen; in this case, there is only one physical barrier, which is made of steel. This test characterizes the ultrasound signal variation by temperature variation between 40 °C and 140 °C. For the temperature range from 40 to 100 °C, S21 increased by approximately +0.71 dB. After 100 °C, from 101 to 140 °C, S21 decreased by −1 dB.

The second test, which uses the Conf.2 configuration and the SP.2 specimen, has three physical barriers between the ultrasonic transducers: two steel and an acoustic fluid barrier. This test is to understand both the transmission of the signal under the condition of multiple layers and the consequence of the communication of the transducers due to their misalignment. Therefore, it selects which UTs were used. Five were fixed to the left plate, three to the left plate, three were fixed to the right plate, and a 100 mm layer of water separated the two plates. This UT organization allowed them to be coupled to have a horizontal alignment of 100%, 40%, 12.5%, and 0%, with 100% completely aligned and 0% completely misaligned. For 100%, the transmission was aligned with a physical barrier, and the signal strength of the S21 was measured at −13 dB; this level is considered unacceptable. However, regarding communication and data transmission, these results are considered favorable for multi-layer data transmission.

For the tensile test near the yield strength, our Test Model 3 uses specimen SP.2 and configuration Conf.1. In this case, the test is conducted without the influence of temperature. The specimen is carefully and gradually stressed up to almost 86% of its yield strength, resulting in a strain of 1250 μm/m. This strain causes a variation in S21 of approximately +0.7 dB. Even with the high deformation, the communication between the UTs remained uninterrupted. This finding is significant, as it indicates that even mechanical strain near the yield strength does not cause detachment in the transducers and, therefore, does not significantly influence the S21 parameter.

Test Model 4 was the last test performed, a test that combines the actuation of mechanical deformation and temperature variation. With deformations up to 125 μm/m and temperatures up to 140 °C, the ultrasonic communication is also not interrupted and has variations of 0.2 dB for just the deformation (this result is consistent with the Model 3 test) and of 1.4 dB combining the deformation with temperature.

Except for UT misalignment, the ultrasonic feedback remains stable for all the interferences we studied. Whether these are temperature variation, adding multiple barriers, mechanical deformation, or combining mechanical deformation with temperature variation, the feedback remains consistent. Even with a low signal and up to 40% of intersections, ultrasonic communication persists. It is important to note that transmission interruption is rare, only happening with 12.5% and 0% of horizontal non-alignment.

## Data Availability

Data are contained within the article.

## Abbreviations

**UT**	Ultrasonic Transducer
**VNA**	Vector Network Analyzers
**PZT**	Piezoelectric Ceramic
**CW**	Continuous Wave
**NDT**	Non-Destructive Testing
**SHM**	Structural Health Monitoring
**TOF**	Time of Flight
